# Fabrication and Characterization of 3D Micro- and Nanoelectrodes for Neuron Recordings

**DOI:** 10.3390/s101110339

**Published:** 2010-11-17

**Authors:** Maria Dimaki, Patricia Vazquez, Mark Holm Olsen, Luigi Sasso, Romen Rodriguez-Trujillo, Indumathi Vedarethinam, Winnie E. Svendsen

**Affiliations:** Department of Micro- and Nanotechnology, Technical University of Denmark, DTU Nanotech, Building 345E, Kgs. Lyngby, Denmark; E-Mails: Patricia.Vazquez@nanotech.dtu.dk (P.V.); Mark.Olsen@nanotech.dtu.dk (M.H.O.); Luigi.Sasso@nanotech.dtu.dk (L.S.); Romen.Trujillo@nanotech.dtu.dk (R.R.-T.); Indumathi.Vedarethinam@nanotech.dtu.dk (I.V.); Winnie.Svendsen@nanotech.dtu.dk (W.E.S.)

**Keywords:** 3D electrodes, fabrication, neuron differentiation on chip

## Abstract

In this paper we discuss the fabrication and characterization of three dimensional (3D) micro- and nanoelectrodes with the goal of using them for extra- and intracellular studies. Two different types of electrodes will be described: high aspect ratio microelectrodes for studying the communication between cells and ultimately for brain slice recordings and small nanoelectrodes for highly localized measurements and ultimately for intracellular studies. Electrical and electrochemical characterization of these electrodes as well as the results of PC12 cell differentiation on chip will be presented and discussed.

## 1. Introduction

Recording electrical or chemical signals from cells can provide a lot of information about how they respond to different stimuli, how they communicate with each other and how they function in general. This kind of understanding of cell dynamics can ultimately help modern medicine treat diseases for which little can be done today, e.g., Parkinson’s, spinal cord injuries, and even cancer [1,2].

Traditionally, recording of cell signals has utilized planar (2D) electrodes. For the recording of electrical signals from neurons, for example, microelectrode arrays (MEAs) are commonly used [3–8]. MEAs are usually circular electrodes of some tens of microns in diameter on top of which the cells are cultured. Signals are only recorded from the cells that lie on the surface of a specific electrode. The quality of the recorded signal is proportional to the surface area of the electrode which is covered by the cell. This is why planar MEAs fabricated to record signals from single cells should have a diameter of around 10–20 μm, corresponding to the size of the cells whose signals are to be recorded. The contact properties between the cell and the electrode are also important; poor contact decreases the signal [9,10] and increased electrode surface roughness increases the signal [11].

In terms of recording chemical signals from cells, electrochemistry is utilized [12]. This has also been carried out with planar electrodes modified with enzymes in order to record a particular response, e.g., glucose oxidase for measuring glucose release [13]. The surface area of such electrodes is also important in this case in terms of sensitivity [14].

As electrical signals from cells are small, on the order of microvolts, they can easily be buried in noise. It is therefore imperative to improve the signal to noise ratio (SNR) of such recordings. This can be achieved by e.g., reducing the impedance of the electrodes. It has been shown that 3D electrodes are promising candidates for improving the SNR, e.g., in [15], where 60 μm high 3D electrodes were fabricated in glass wafers and used as a MEA system in place of the 2D electrodes. Assuming a conical shape of the electrode the gain in surface area between a 3D electrode with a radius r and height h and a planar electrode of the same radius is $\sqrt{\left(h^{2}+r^{2}\right)/r}$, which in turn means that the signal recorded from a 3D electrode will be larger, purely due to the larger surface area. The higher the aspect ratio of the 3D structures, the bigger the gain in surface area. The conical model for the electrodes underestimates their surface area. On the other hand, this generalization is only valid if one assumes that the cell completely covers the 3D electrode as it does with the 2D electrode.

Moreover, when signals from tissue slices are to be recorded, the existence of a dead cell layer between the electrode and the firing neurons further deteriorates the quality of the signals. In these cases it is imperative to have tall electrodes that can be pushed through the tissue and record the signals at a closer distance.

The realization that 3D electrodes can offer better SNR for cell or tissue recordings fueled the interest for fabricating novel 3D electrode structures. A variety of fabrication techniques (like wet and dry etching, electrochemical deposition) were used for the fabrication of both tall [15–19] and small [20] electrodes for measuring on tissue slices and individual neuron cells.

The electrodes developed in [15], along with similar electrodes sold by Multi Channel Systems MCS GmbH have been commercialized and have been used in many publications demonstrating relevant biological measurements on tissue slices [21–24]. Their ability to penetrate the slices and come closer to the firing neurons as well as the improved SNR has been of great importance in the field and therefore they find excellent applications in studies of brain slices.

Smaller electrodes are not as easy to fabricate, especially not with conventional fabrication methods. The main reason for fabricating small electrodes is for intracellular measurements, where tips of the order of 100–200 nm are necessary in order to keep the cells alive. Their small dimensions can also offer a great spatial resolution, though integration of all connecting wires for contacts to the outside world then needs to be done by CMOS technologies.

In this paper we will present the fabrication, characterization and testing of two types of 3D electrodes: 1) ca. 60 μm tall electrodes with a base of 40 μm in diameter intended for measurements in tissue slices and 2) up to 5 μm tall electrodes, about 3 μm in base diameter and sub 200 nm tips, fabricated using conventional microfabrication techniques and intended for intracellular measurements. Finally, differentiation of PC12 cells on the chips will be demonstrated, proving the chips’ biocompatibility.

## 2. Experimental Section

### 2.1. Fabrication of Tall Electrodes

**Figure 1.**
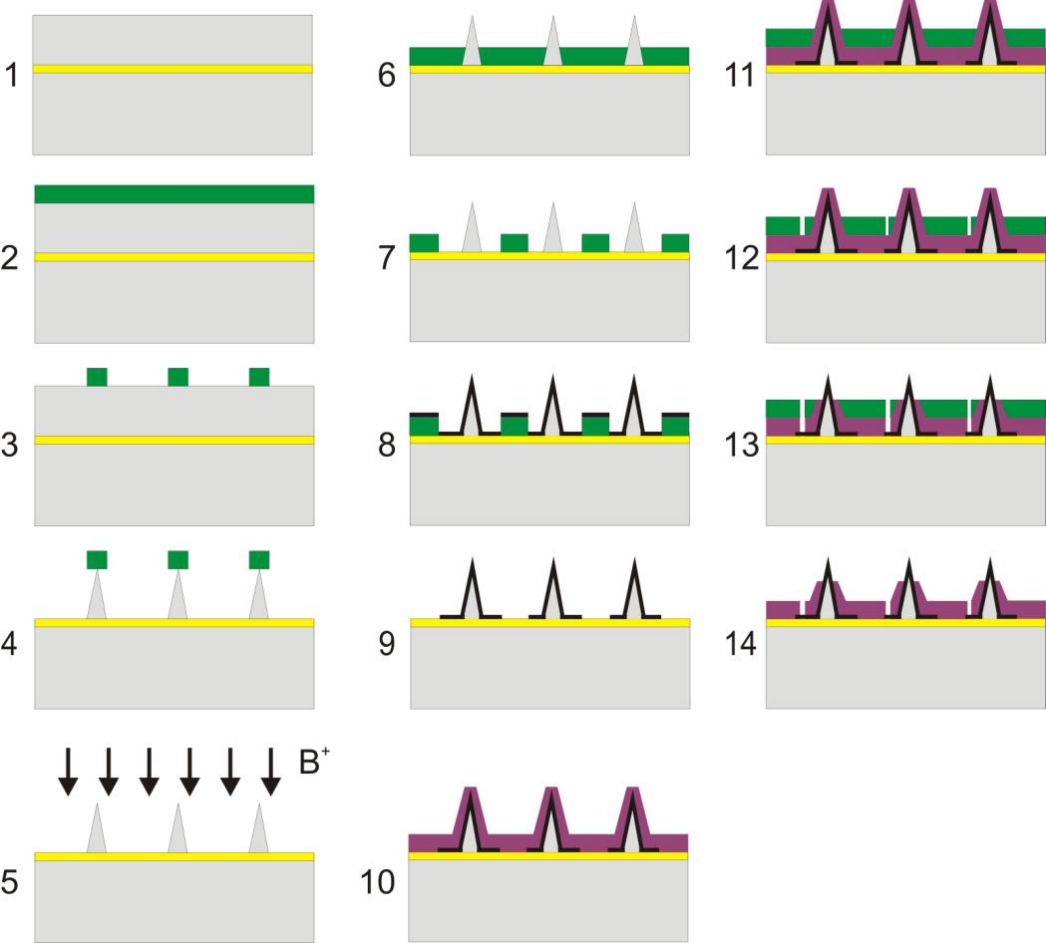
The fabrication process for the tall 3D electrodes.

The electrodes are fabricated on a Silicon On Insulator (SOI) wafer with a 500 nm buried silicon oxide layer and a top layer of about 65 μm. The top layer is made of highly doped silicon for higher conductivity. A cross-section sketch of the fabrication process is shown in Figure 1.

Briefly, starting with a SOI wafer with a top silicon layer of ca. 65 μm (step 1), a 4.2 μm thick layer of photoresist is spun on the wafer (step 2). Next the resist is exposed and developed to mask the electrodes (step 3). The electrodes are then etched on the top silicon layer using a combination of dry anisotropic and isotropic etching with an Advanced Silicon Etcher (ASE) (step 4). The best combination found was 163 sec of anisotropic etching followed by 110 sec of isotropic etching. Twelve of these etching cycles are used for making the electrodes.

Once etched, the electrodes are doped with boron using a deposition oven, in order to increase their conductivity (step 5). This step can be skipped if a highly doped top silicon layer is used. The photoresist is then spun (step 6) before photolithography to pattern the connection wires and bonding pads, as well as metalize the electrodes (step 7), followed by deposition of metal (20 nm Ti and 200 nm Pt or Au) (step 8), lift-off (step 9), deposition of 500 nm silicon nitride by Plasma-Enhanced Chemical Vapour Deposition (PECVD) (step 10), then spinning of photoresist (step 11), photolithography to pattern the contact to the bonding pads and the electrode openings (step 12), etching of silicon nitride (step 13) and finally removal of photoresist (step 14). In step 13 holes are opened through the nitride layer using a Reactive Ion Etch (RIE) on top of the electrodes and at the bonding pads for contact to the measurement equipment.

The metallization in reality is not as good, as is shown schematically in step 8 in Figure 1, as the steepest places on the electrodes are not covered with metal or are only covered by a thin layer [25,26]. That is why the doped silicon is required; to secure a conductive path to the top of the electrodes. As the electrodes are doped, the metallization of the tall 3D structures does not have to be perfect or complete.

### 2.2. Fabrication of Small Electrodes

**Figure 2.**
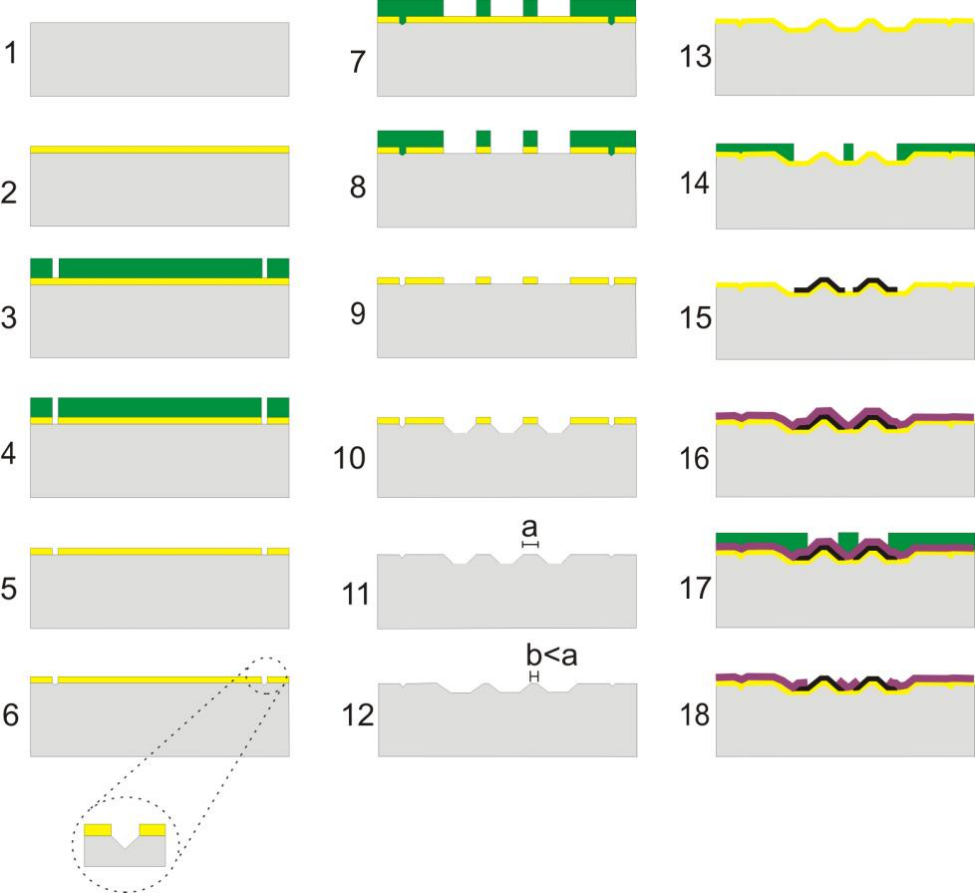
The fabrication process for the small electrodes.

A sketch of the fabrication process can be seen in Figure 2. The small electrodes are fabricated starting with a normal single polished silicon wafer (step 1) on which a 500 nm layer of silicon oxide is grown (step 2), followed by spinning of 1.5 μm photoresist, and photolithography of (110) alignment structures (step 3). The mask is aligned to the 110 direction so that a controlled uniform etch with KOH can be achieved. This process is based on the work described in [27]. After etching of oxide with RIE or Buffered HF (BHF) (step 4) and removal of photoresist (step 5) the alignment structures are etched with KOH (step 6), the photoresist is spun and the electrodes patterned (step 7), followed by RIE or BHF etch of oxide (step 8) and removal of photoresist (step 9).

The electrode structures are fabricated by etching in silicon with an 80 °C warm KOH solution (28%) for 4.5 minutes (step 10) and removal of oxide (step 11). This process is followed by a maskless isotropic etch in an ASE for 30 sec to 2 min, which serves to sharpen the electrodes further (step 12). After this the electrodes are electrically isolated by deposition of 500 nm silicon dioxide using a PECVD process (step 13). This step should ideally be done by thermal silicon dioxide growth in an oven, as the PECVD oxide has worse quality. The isolation step is followed by spinning of resist (step 14) and patterning of metallization of electrodes and connection wires (100 nm platinum patterned with a lift-off process) (step 15), passivation by deposition of 200 nm of silicon nitride with a PECVD process (step 16) and patterning of openings of the passivation layer (step 17). Finally, the nitride is etched from the electrode area using a RIE to make them electrically active (step 18).

### 2.3. Impedance Characterization

**Figure 3.**
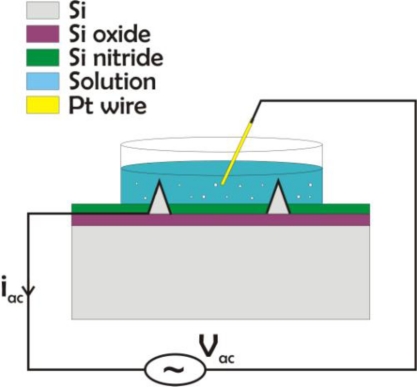
Set up for impedance measurements. The analyzer sets an AC voltage between one electrode and a larger platinum wire in the solution and measures the current at different frequencies.

For the electrical characterization of the electrodes an impedance analyzer HP4294A was used. This can measure impedance in the range of 40 Hz to 110 MHz. The experimental setup is shown in Figure 3.

The characterization was done in a 1% NaCl solution following the example of [15]. For the small 3D electrodes measurements were additionally taken in de-ionized (DI) water as well as a 0.1% NaCl solution. The impedance of the tall electrodes was measured in the biologically relevant range of 40 Hz to 100 kHz. As the impedance of the small electrodes is expected to be large, the frequency range for the measurements was from 100 Hz to 80 MHz. The input voltage amplitude was 100 mV in both cases. Impedance measurements were also done at 10 mV but apart from a higher noise at the lowest frequencies in this case, the impedance values were identical for 10 mV and 100 mV. Therefore 100 mV were chosen as the voltage amplitude to avoid high noise levels. Frequencies down to 1 Hz would also be relevant for this characterization considering the application, but due to the experimental equipment these were not possible to test.

### 2.4. Electrochemical Characterization

Using a Reference 600 potentiostat/galvanostat/ZRA (Gamry Instruments, Warminster, PA, USA) Polyaniline (PAni) was electrodeposited on selected small electrodes by varying the potential from 0 to 1 V at a potential sweep rate (PSR) of 100 mV/s for 25 cycles in a 1.0 M HCl solution containing 1.0 M of the aniline monomer using an Ag/AgCl (1.0 M KCl) reference electrode. The same instrument was used afterwards for getting cyclic voltammograms from the PAni covered electrodes at PSR from 50 to 300 mV/s.

### 2.5. Energy Dispersive X-Ray (EDX) Analysis

EDX analysis (Oxford Inca EDX system, Oxford Instruments, UK) on the small electrodes was done inside a Scanning Electron Microscope (SEM) from FEI Corporation in order to check the electrodeposition of PAni on the selected electrodes and whether the silicon nitride layer was properly removed during the nitride etch (step 18, Figure 2). Spectra were taken at 15 kV on three locations: 1) An electrode location that had not been contacted during electrodeposition of PAni 2) An electrode location that was contacted during electrodeposition of PAni and 3) A test electrode for which the nitride layer had not been removed during the nitride etch step.

### 2.6. PC12 Cell Culture and Differentiation

Untreated T25 flasks (Nunc, Thermo Scientific, Denmark) were coated with laminin (20 μg/mL, Sigma Aldrich, Denmark) in 1X Phosphate Buffered Saline (PBS) (Sigma Aldrich, Denmark) and left overnight. Later, excess laminin was removed and the flasks were washed twice with sterile water (Sigma Aldrich, Denmark). The Dulbecco’s Modified Eagle medium/Ham’s Nutrient Mixture F12 supplemented with 10% fetal bovine serum, 10% horse serum, 100 Units/mL penicillin and 100 μg/mL streptomycin and 25 mM HEPES (referred to as DMEM/F12 from now on) was added in the flasks and placed at 37 °C in humid atmosphere containing 95% air and 5% CO_2_.

A vial containing approximately 4 × 10^6^ cells (PC12-pheochromocytoma of rat adrenal medulla, DSMZ GmbH) was thawed and transferred to the above flasks and placed in the incubator. After 2 days the media was removed and replaced with DMEM/F-12 supplemented with 100 ng/mL Neurite Growth Factor (NGF). The cell media was changed every 2 days. After 5 days, the cells were detached by adding 0.05% trypsin-EDTA (Sigma Aldrich, Denmark) at 37 °C for 3 min. The cell suspension was transferred to a 10 mL Falcon tube with fresh media and centrifuged at 1,100 rpm for 3 minutes. The supernatant was removed and the pellet was resuspended with media at 1 × 10^6^ cells/mL. The centrifugation process and the resuspension of the cells in fresh media are done in order to remove the trypsin.

The cells were then seeded in a Petri dish containing the electrode chip and supplemented with DMEM/F-12 with NGF. Before seeding the cells, the electrode chips were sterilised with acetone at 70 °C for 5 min followed by three times wash in 70% ethanol for 5 min. The chips were washed with 1× PBS three times and then coated with laminin, as previously described. After 32 hrs, two thirds of the culture medium was removed, the culture was fixed on the surfaces with 4% paraformaldehyde fixative solution (4% of paraformaldehyde in 0.1 M PBS) for 15 min and the fixative solution was removed. The sample was analyzed using a ZEISS Axio microscope with 20× and 50× magnification.

For Scanning Electron Microscope (SEM) imaging, the cells were fixed in 2% glutaraldehyde in 0.1 M PBS for an hour. The sample was rinsed in 0.1 M PBS for 15 minutes at 4 °C and washed with deionized water for 5 minutes. The sample was dehydrated with increased percentages of ethanol (50, 60, 70, 80, 90, and finally 100%) and freeze dried overnight.

## 3. Results and Discussion

### 3.1. Fabrication of Tall Electrodes

**Figure 4.**
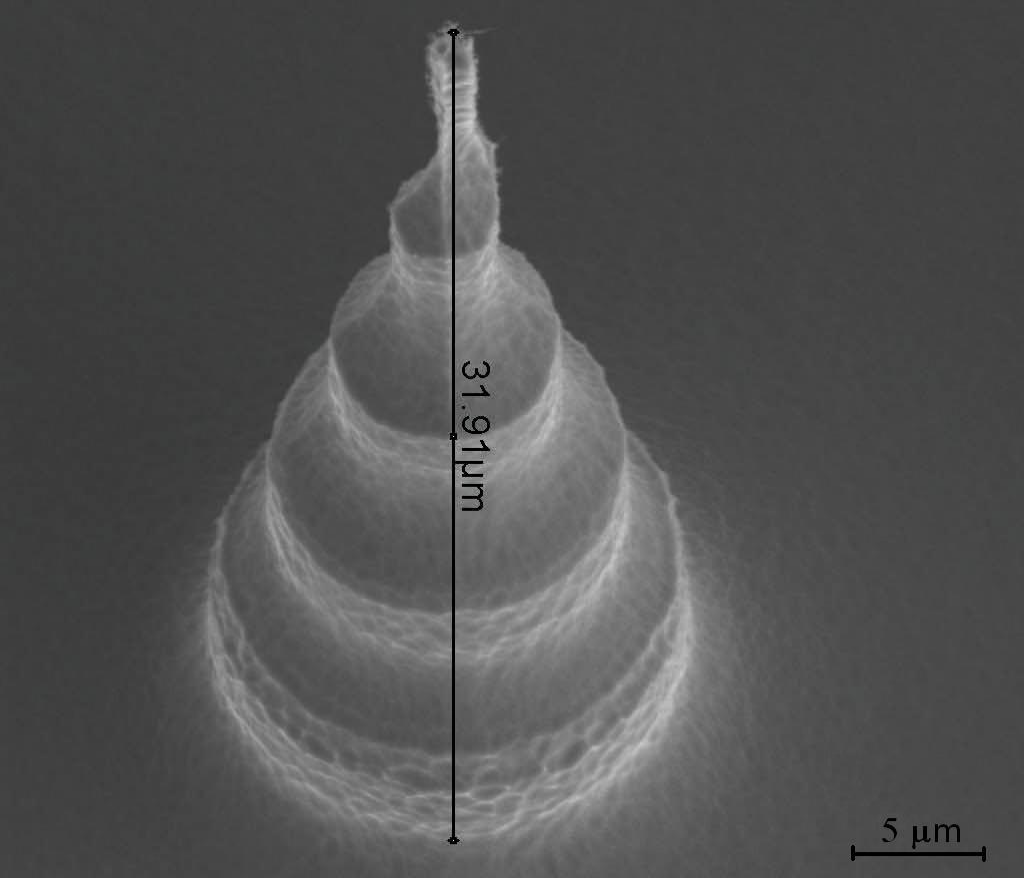
The measurement of the height of the pillar should be corrected with the tilted angle with which the picture was taken in the SEM (30°), giving a value of 63.8 μm.

The fabrication of the tall electrodes gives them a characteristic scalloped shape that increases their surface area as compared to a 2D electrode or a conical 3D electrode (Figure 4).

**Figure 5.**
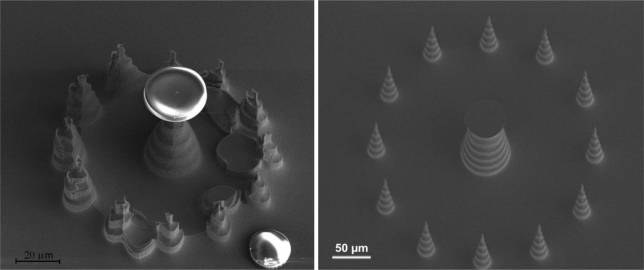
Left: The sacrificial pillars (outside circle) are destroyed during the process but the main electrode remains intact and obtains a scalloped profile. The round discs on top of the main pillar and on the substrate are the resist masks used for the etching. Right: The process can be changed so the sacrificial pillars remain on the chip. In this case they can be used as electrodes themselves enabling localized electrochemical or electrophysiological measurements.

As mentioned earlier, this is desirable for improving the quality of the signals recorded. Moreover, the fabrication process is much easier to optimize and control. The electrodes are surrounded by pillars that fulfill two purposes: first, they protect the main structure from being damaged during the etch process; in this case the sacrificial pillars disappear during the creation of the electrodes due to the aggressiveness of the process, as it is shown in Figure 5 (left). At this point in the process the sacrificial pillars are still on the chip but highly deformed. Secondly, the pillars can be kept and shaped like electrodes surrounding the big electrode in the middle. Such a configuration can be used for localized electrochemical measurements, where the sacrificial pillars act as the working electrode and the now large middle electrode acts as the reference or counter electrode. The same pillars can be used as electrodes for electrophysiological measurements providing multiple signals from the same area in the slice. An image of the finished configuration in this case is shown in Figure 5 (right).

The dry etch based fabrication process used also allows for a smaller pitch between the electrodes while maintaining the large height needed for tissue slice measurements. Due to the cake-like shape of the electrode they also have a larger surface area than isotropically wet etched structures, which is needed for achieving a good signal-to-noise ratio.

### 3.2. Fabrication of Small Electrodes

**Figure 6.**
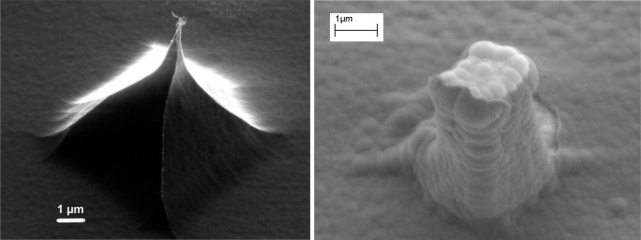
Left: An electrode after the KOH etch and ASE isotropic etch. The image is taken at a 45° angle. Right: A finished pillar with 500 nm PECVD oxide for isolation, 100 nm Pt layer for metallization and 200 nm nitride layer for passivation. The nitride layer has been opened at the top of the pillar for establishing electrical contact with the cells.

As shown in Figure 6 (left), it is possible to fabricate small 3D electrodes with sub 200 nm tips and heights of 3–5 μm in silicon using standard microfabrication techniques. The sub 200 nm tips are of course enlarged by the deposition of the subsequent layers. Thus the pillar shown in Figure 6 (right) is significantly larger, with a tip of about 1 μm. This can, however, be reduced further by using a thermally grown silicon oxide layer instead of the PECVD one shown in Figure 6 (right). The advantage of this is that it has a better quality and therefore a significantly thinner layer can be used for isolation, e.g., 200 nm instead of 500 nm. That would reduce the pillar tip by at least 600 nm. Moreover, as thermally grown oxide consumes the underlying silicon, further sharpening of the silicon pillar can be achieved in this step.

The reproducibility of this fabrication process is greatly dependent on the KOH bath age and previous load [28], *i.e.*, how many μm of silicon have been etched with the same bath before this process. The uniformity is mostly a matter of photolithography as well as micromasking effects caused by wafer impurities and inadequate stirring in the tank. Both of these issues arose in our case with uniformity being the largest one. From SEM images, the wafer uniformity was estimated to be from 50 to 80%.

The fabrication process presented allows us to fabricate electrodes with a pitch down to at least 5 μm, which provides a larger spatial resolution for neuron action potential propagation studies than what is the norm today. For non C-MOS chips, however, requirements for space for the connecting wires push the minimum spacing between electrodes to about 30–40 μm.

Although the tip size of the finished electrode is currently not small enough to allow penetration of the cell membrane without killing the cell [29], at least not for long term measurements, further shrinking of the tip size as described above is possible and would result in electrodes with the proper dimensions. Moreover, the silicon oxide isolation layer can be omitted completely from the fabrication process by using SOI wafers in the same way as for the large pillar electrodes described in Section 2.1. This way the total thickness of the deposition material at the electrode tip can be kept at the thickness of the metal layer.

### 3.3. Impedance Characterization

**Figure 7.**
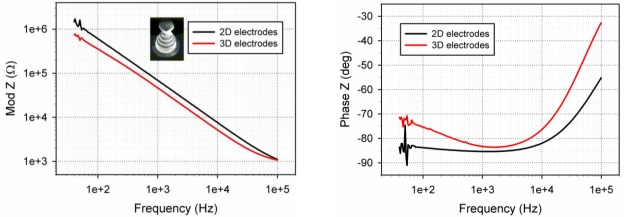
Plot of average impedance of four large 3D electrodes *vs.* four 2D electrodes of 40 μm diameter electrodes (left: impedance modulus, right: phase). The electrodes were submerged in a 1% NaCl solution.

For the tall electrodes five measurements were conducted on identically fabricated electrodes. Moreover, four 2D electrodes of the same dimensions and at the same locations on the chip as the 3D electrodes were examined. All of the tested electrodes were functional and 4 out of 5 exhibited impedance that was smaller than the impedance of the corresponding 2D electrode. These measurements were done in the range of 40 Hz to 100 kHz and the average values are shown in Figure 7. The electrical impedance of the tall electrodes is theoretically expected to be 3.8 times smaller than the corresponding 2D electrodes. With the exception of one electrode, the impedance of the 3D electrodes is on average 1.5–1.7 times smaller than that of the 2D electrodes. We note that the electrodes are a bit different than those shown in Figure 4, as can be seen in Figure 7 (left), where the tip diameter is around 10 μm and also features an undercut. The surface area of these new structures is enhanced compared to electrodes of conical shape, but they are not as good in terms of penetrating tissue slices. However, they have the advantage of being able to “automatically” anchor the tissue slices as the undercut functions as a sort of hook, holding the slices in place without the need for weights to hold the slice down.

The results described in Figure 7 are in agreement with the results presented in [15], as the impedance values achieved with the presented electrodes are of the same order of magnitude as those in [15] and in fact a bit smaller on average, probably as a result of the increased surface area. Compared to the impedance recorded by the 2D electrodes one can see that the 3D electrodes are better, though not as much as theoretically expected. This result is peculiar; however, one should keep in mind that the comparison is not really fair, as the 3D electrodes are made of highly doped silicon as the conducting material as opposed to the gold or platinum 2D electrodes. This alone results in a higher impedance, so the fact that the impedance is smaller for the 3D electrodes and at least as good as that of the platinum covered electrodes described in [15] is very encouraging.

**Figure 8.**
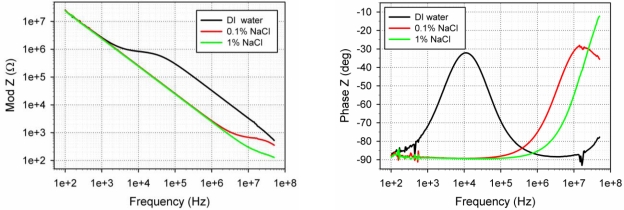
The impedance characterization of the small 3D electrodes in DI water, 0.1% NaCl and 1% NaCl. Left: impedance modulus, Right: Impedance phase.

The impedance of the small 3D electrodes in DI water, a 0.1% and 1% NaCl solution is shown in Figure 8. The plotted curves are average values taken from 5 different electrodes on the same chip. The figure shows that although there is no difference in the electrode impedance in the 3 different solutions in the low frequency range, there is a clear difference at higher frequencies, where the monitored impedance is that of the solution between the monitored electrode and the counter electrode (the large platinum wire of Figure 3). This suggests that the recorded impedance is indeed that of the electrodes and not just due to parasitic capacitances through the substrate.

Comparing Figure 7 and Figure 8 it can be seen that the impedance of the small electrodes is relatively large, in agreement with previous work [30] and theory. However, it is still possible to measure a difference when submerging the electrodes in different solutions. Considering that the surface area of the small electrodes is not more than 1–10 μm^2^, about 400 times smaller than that of the large electrodes, it is not surprising that the impedance is about 2 orders of magnitude larger than that of the tall electrodes. We note that the phase of the impedance is around −90° but starts moving towards zero at about 100 kHz to 1 MHz, depending on the solution. The change happens at lower frequencies for the tall electrodes, at around 5–10 kHz, as is the case for commercial systems. The large impedance of the small electrodes would make electrophysiological measurements with these quite difficult; this can however be improved by depositing platinum black on the electrode openings. This will increase the surface area of the electrodes due to its fractal/porous structure and therefore reduce the impedance.

### 3.4. Electrochemical Characterization

**Figure 9.**
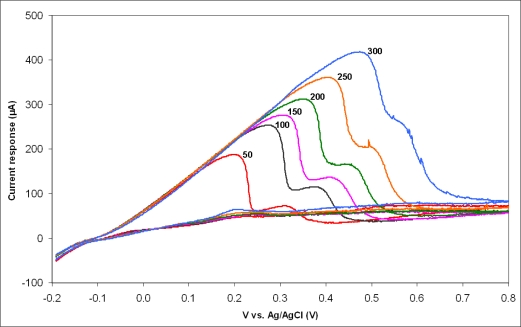
Cyclic voltammograms from the small ele**c**trodes after the electrodeposition of a polyaniline film in a 1.0 M HCl solution using an Ag/AgCl reference electrode, for potential sweep rates of 50, 100, 150, 200, 250 and 300 mV/s.

The electrochemical response at six different PSRs is shown in Figure 9. There is a clear signal from the PAni covered electrodes and the behaviour is irreversible. The irreversibility is established not only by the lack of the reduction peak but also because the oxidation peak potential shifts towards higher voltages with increasing PSR and because the peak current, though increasing with increasing PSR, is not proportional to the square root of the PSR. The two anodic peaks observed in the positive forward scan of the voltammogram correspond to the transition of PAni from leucoemeraldine (fully reduced) to emeraldine (partially oxidized) and from emeraldine to pernigraniline (fully oxidized). These results are comparable to the cyclic voltammetric response of PAni grown on several conductive substrates [31]. The fact that the electrodes are electrochemically active is a good indication that the nitride layer has successfully been removed from the electrode openings. Moreover, the results show that the deposition of polymers on top of the small electrodes is possible, which opens up for a number of possibilities to use these electrodes as electrochemical sensors. Indeed, the trapping of enzymes within electrodeposited polymers is a well established technique first shown in [32] and one that has the ability of producing highly localized and selective sensors. Combined with the possibility of addressing individual cells with the small electrodes this could allow for the electrochemical recording of metabolic products from neurons on a single cell level.

### 3.5. EDX Analysis

**Figure 10.**
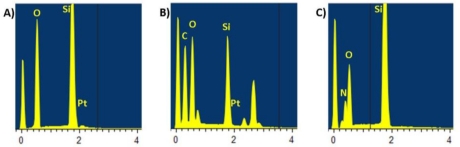
EDX analysis spectra of **(A)** a small electrode at the end of the fabrication process after the nitride etching step, **(B)** a small electrode after an electrochemical polymerization of aniline, **(C)** a small electrode structure present in the wafer but that has not undergone a nitride etching step. Spectra were taken at 15 kV, units on the horizontal axis are keV, units on the y axis are arbitrary units reflecting the amount of the material present.

The results of the EDX analysis are shown in Figure 10. A peak corresponding to Pt can be seen on an electrode where the nitride layer has been removed, and indeed no peak corresponding to N is visible (Figure 10A). However, a test electrode with no platinum and the nitride layer not removed shows this peak (Figure 10C). Moreover, an electrode with platinum, the nitride layer removed and PAni electrodeposited on it has peaks corresponding to C and Pt but no peak for N (Figure 10B). The EDX analysis therefore confirms that the nitride passivation layer has been opened at the top of the electrodes as intended. It also shows that the electropolymerization of aniline (electrodeposition of PAni) is localized on the contacted electrodes, which opens up exciting possibilities for selective functionalization of the electrodes.

### 3.6. PC12 Cell Culture

The final measurements carried out aimed at investigating the toxicity of the electrode substrates. Here we not only investigate the toxicity of the silicon nitride substrate that is used as a passivation layer but the toxicity of the entire chip after it has undergone all the different fabrication processes. Despite cross contamination rules and code of conduct in the cleanroom, commonly used steps like resist spinning, developing and stripping as well as the placement of the wafers in the various etchers or deposition machines can in principle contaminate the chip with metallic particles or other impurities. From previous experience this can often have a lethal effect on the cells, even though perfectly biocompatible materials and processes have been used for the fabrication. After 32 hours of culture images of PC12 cells on the electrode substrates were taken both with an optical microscope as well as a SEM.

**Figure 11.**
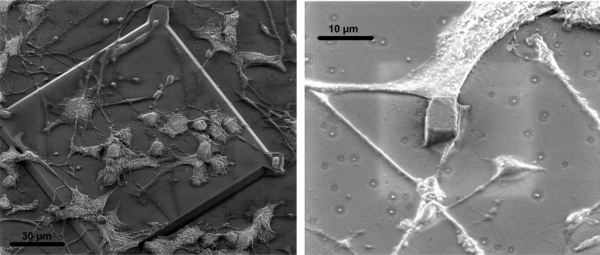
PC12 cells growing on the small electrode chips. Left: cells on a test area on the chip. Right: the main body of one PC12 cell and an axon on the side of a pillar.

As can be seen in Figure 11 the neurons not only survive, but attach on the surface and differentiate, *i.e.*, spread axons. In a subsequent experiment the neurons survived and differentiated on chip for more than eight days before measurements were attempted. This is a good indication of the biocompatibility of the substrates and also shows that it is possible to coat the PECVD silicon nitride surfaces with laminin for neuron differentiation, in accordance to available literature [33–35].

In the case of the small electrodes the cells are attaching both with their main body and with their axons to them, which means that it will be possible to measure propagation of electrical signals and even penetrate the cell body for intracellular measurements with a proper penetration mechanism.

**Figure 12.**
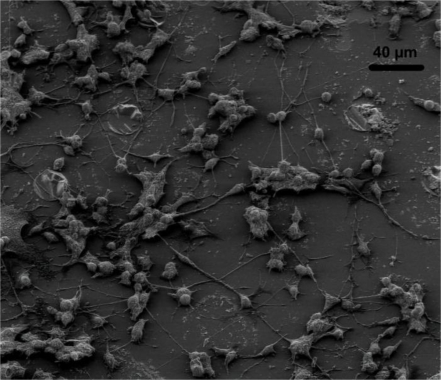
Image of differentiated neurons on the tall electrode substrate (no pillars). This substrate has been though the same process as the substrates containing the pillars and is only used for biocompatibility studies.

The tall electrodes are meant to be used with tissue slices, but neurons were also cultured on them in order to check the biocompatibility of the chips after the complete fabrication process. Due to SEM unavailability only optical microscopy images were taken for the tall electrodes. These show that the cells are growing and differentiating on the entire chip surface but the images lack the detail of the SEM images and are therefore not shown here. In Figure 12 SEM images of cells growing on substrates that had undergone the same fabrication processes as the tall 3D electrodes but without the pillars are shown for demonstration purposes.

## 4. Conclusions

We have shown that we are able to fabricate functional high aspect ratio and small sub 200 nm tip 3D electrode structures for the investigation of cell and tissue cultures using standard microfabrication techniques. The 3D electrodes have been characterized both electrically and electrochemically.

The tall 3D electrodes show a smaller impedance compared to size equivalent 2D electrodes, though the reduction is not as high as expected from theory. This may be due to the fact that the electrodes are made of doped silicon, which has a lower conductivity than gold or platinum. Considering this, the resulting impedance is indeed quite comparable to and even slightly better than the values found for commercial systems.

The small 3D electrodes impedance is relatively large, about two orders of magnitude higher than that of the tall structures. This result is however not surprising, considering their small surface area. Whether or not electrophysiological measurements can be performed with these electrodes remains to be seen, however, we have shown that they are electrochemically active, with an irreversible behaviour. This makes them good candidates for electrochemical detection of e.g., metabolic products from neurons, especially by trapping enzymes within functionalizing electrodeposited polymer layers.

Finally, we have shown that PC12 cells can differentiate on the chips with the 3D electrodes and thrive for at least eight days. Future work on these systems will involve the optimization of the fabrication processes and the recording of electrical and electrochemical signals from neuron cells in culture and in tissue slices.
